# MicroRNA-193b Enhances Tumor Progression via Down Regulation of Neurofibromin 1

**DOI:** 10.1371/journal.pone.0053765

**Published:** 2013-01-15

**Authors:** Michelle Lenarduzzi, Angela B. Y. Hui, Nehad M. Alajez, Wei Shi, Justin Williams, Shijun Yue, Brian O’Sullivan, Fei-Fei Liu

**Affiliations:** 1 Ontario Cancer Institute, University Health Network, Toronto, Canada; 2 Department of Medical Biophysics, University of Toronto, Toronto, Canada; 3 Stem Cell Unit, Department of Anatomy, College of Medicine, King Saud University, Riyadh, Saudi Arabia; 4 Radiation Medicine Program University Health Network, Toronto, Canada; 5 Department of Radiation Oncology, University of Toronto, Toronto, Ontario; University of Illinois College of Medicine, United States of America

## Abstract

Despite improvements in therapeutic approaches for head and neck squamous cell carcinomas (HNSCC), clinical outcome has remained disappointing, with 5-year overall survival rates hovering around 40–50%, underscoring an urgent need to better understand the biological bases of this disease. We chose to address this challenge by studying the role of micro-RNAs (miRNAs) in HNSCC. MiR-193b was identified as an over-expressed miRNA from global miRNA profiling studies previously conducted in our lab, and confirmed in HNSCC cell lines. *In vitro* knockdown of miR-193b in FaDu cancer cells substantially reduced cell proliferation, migration and invasion, along with suppressed tumour formation *in vivo*. By integrating *in silico* prediction algorithms with *in vitro* experimental mRNA profilings, plus mRNA expression data of clinical specimens, neurofibromin 1 (NF1) was identified to be a target of miR-193b. Concordantly, miR-193b knockdown decreased NF1 transcript and protein levels significantly. Luciferase reporter assays confirmed the direct interaction of miR-193b with NF1. Moreover, p-ERK, a downstream target of NF1 was also suppressed after miR-193b knockdown. FaDu cells treated with a p-ERK inhibitor (U0126) phenocopied the reduced cell proliferation, migration and invasion observed with miR-193b knockdown. Finally, HNSCC patients whose tumours expressed high levels of miR-193b experienced a lower disease-free survival compared to patients with low miR-193b expression. Our findings identified miR-193b as a potentially novel prognostic marker in HNSCC that drives tumour progression *via* down-regulating NF1, in turn leading to activation of ERK, resulting in proliferation, migration, invasion, and tumour formation.

## Introduction

Head and neck squamous cell carcinoma (HNSCC) is the 6^th^ most common cancer worldwide, with ∼650,000 new cases diagnosed, and ∼350,000 deaths annually [1], [2]. With the majority of patients presenting with locally advanced disease, and despite improvements in treatment approaches, the 5-year survival rates of 40–50% have not significantly improved in the past decades [3], underscoring an urgent need to better understand the molecular mechanisms underlying the biology of this disease.

We have chosen to address HNSCC biology through the lens of micro-RNAs (miRNAs), an endogenous class of non-coding RNAs that negatively regulate gene expression through translational repression or degradation of mRNAs targets in a sequence specific manner [4]. Since their initial identification in nematodes in 1993, miRNAs have been described to regulate a number of biological processes, including cancer [5], [6]. *In silico* algorithms predict that miRNAs control one third of protein encoding genes, rendering them as one of the largest classes of gene regulators [7]. In recent years, aberrant miRNA expression has been recognized to enhance cancer progression *via* their mRNA targets [8], [9]. In this study, we report the over-expression of miR-193b in HNSCC, based on our global miRNA profiling of HNSCC cell lines, our comprehensive miRNA profiling study of relapsed *vs*. non-relapsed primary HNSCC tissues [10], and a study by Avissar *et al.* [11]. In turn, we have identified neurofibromin 1 (NF1) as a target of miR-193b, which drives HNSCC progression *via* ERK activation.

## Materials and Methods

### Ethics Statement

All animal experiments were conducted in accordance to guidelines of the Animal Care Committee at the University Health Network (Toronto, Canada). The protocol was approved by the Animal Care Committee at the University Health Network (Protocol Number: 342.19).

Patient samples were collected from a phase III randomized study (331 participants) of hyperfractionated radiotherapy conducted in 1988 to 1995 [10], [12], with approval from the University Health Network Institutional Research Ethics Board (REB approval # 07-0521-CE). Written or Oral consent could not be obtained from the patients due to the period of our cohort (1988–1995). Therefore, our University Health Network Institutional Research Ethics Board waived the requirement for written informed consent from the participants of this study.

### Cells Lines and Reagents

The human hypopharyngeal HNSCC FaDu cell line was obtained from the American Type Culture Collection (Manassas, VA), and cultured according to the manufacturer’s specifications. The human laryngeal squamous cell lines, UTSCC-8 and UTSCC-42a (kind gifts from R Grénman, Turku University Hospital, Turku, Finland) [13], [14] were maintained with DMEM supplemented with 10% fetal bovine serum (Wisent, Inc) and 100 mg/L penicillin/streptomycin. The normal oral epithelial cells (NOEs) were purchased commercially and cultured in the recommended medium (Celprogen). All cells were maintained in a 37°C incubator with humidified 5% CO_2_, authenticated at the Centre for Applied Genomics (Hospital for Sick Children, Toronto, Canada) using the AmpF/STR Identifier PCR Amplification Kit (Applied Biosystems), and routinely tested for mycoplasma contamination using the Mycoalert detection kit (Lonza Group Ltd).

### Transfection Experiments

The biological effects of miR-193b were investigated using a lock nucleic acid (LNA) probe containing a sequence specific antisense oligonucleotide targeting miR-193b, miRCURY LNA™ microRNA 193b Power inhibitor (Exiqon). A scrambled miRNA sequence, miRCURY LNA^tm^ microRNA Power inhibitors Control A served as a negative control.

### Quantification of miRNA and mRNA

Total RNA was extracted from either cell lines, or primary tissues using the Total RNA purification kit (Norgen), then reverse transcribed using SuperScript II Reverse Transcriptase (Invitrogen Canada) according to specifications. MicroRNA profiles of three HNSCC cell lines (FaDu, UTSCC 42a and UTSCC 8) compared to NOE were generated with a TaqMan Low Density Array (Applied Biosystems) as previously described [10]. The expression of candidate miR-193b targets: PTPRT, IGFBP5, PER2, SARM1, SLC38A3, CASP9, FABP3, DAB21P, APC2, TP53INP1, ST3GAL4, DUSP1 and NF1 were also assessed using qRT-PCR, performed using SYBR Green Master Mix (Applied Biosystems) and the ABI PRISM 7900 Sequence Detection System (Applied Biosystems Inc, Foster City, CA). The primer sequences used in this study are all listed in Table S1A.

### Viability and Clonogenic Assays

The viability of FaDu cells transfected with locked nucleic acid (LNA)-193b was determined using CellTiter 96 Non-Radioactive Cell Proliferation Assay (MTS) (Promega BioSciences), according to the manufacturer’s recommendations. The colony forming ability of FaDu cells transfected with LNA-193b was determined using clonogenic assay as previously described [15]. Briefly, 72 hours after transfection with LNA-scrambled or LNA-193b, FaDu cells were re-seeded in 6-well plates, and incubated at 37°C under 5% CO_2_ for 8–12 days. The plates were then washed and stained with 0.1% crystal violet in 50% methanol, and the number of colonies was then counted. The fraction of surviving cells was calculated by comparison of LNA-193b with LNA-scrambled transfected cells.

### Cell Cycle Analysis

Cell cycle analysis was conducted on FaDu cells after LNA-193b transfection, as previously described [15]. Briefly, cells were collected and washed twice in FACS buffer (PBS/0.5% BSA), re-suspended, then fixed in ice-cold 70% ethanol. After a second wash, cells were re-suspended in FACS buffer containing RNAse A (Sigma, St. Louis, MO, USA) and propidium iodine. Cells were incubated in the dark at room temperature before being analyzed in BD FACScalibur (Becton Dickinson, San Jose, CA, USA) using FL-2A and FL-2W channels. The flow cytometry data were analyzed using FlowJo 7.5 software (Tree Star, San Carlos, CA, USA).

### Cell Migration and Invasion

To assess the migration and invasion potential of FaDu cells after transfection with LNA-193b, BD BioCoat Control 8.0 um PET Membranes, were utilized to measure migration, and BD BioCoat Matrigel™ invasion chambers with a uniform layer of BD Matrigel basement membrane matrix were used to measure invasion. For both the migration and invasion assays, the chambers were placed in a 24-well plate, and 1.5×10^5^ cells in 0.5% serum were added to the top of the chamber, and 20% serum added to the bottom chamber. Twenty-four hours later, inserts were fixed and stained with SIEMENNS DIFF-QUICK stain set (Siemens Healthcare Diagnostics), and the number of migrating and invading cells was counted using a light microscope.

### Tumour Formation Assay

FaDu cells were transfected with LNA-scrambled or LNA-193b. Forty-eight hours later, viable cells were harvested and 2.5×10^5^ cells were suspended in 100 uL of media, and injected intramuscularly into the left gastrocnemius muscle of 8–10 week old female severe combined immunodeficient (SCID) BALB/c mice. Tumour growth was monitored by measuring the tumor plus leg diameter (TLD) two-three times per week; mice were sacrificed once the TLD reached 13 mm as a humane end-point.

### Luciferase Assay

The 3′ UTR of NF1 containing the predicted miR-193b binding site was amplified using AmpliTaq gold DNA polymerase (Applied Biosystems), using the indicated primers (Table S1B). Next, the PCR product was purified, digested with SpeI and HindIII, then cloned downstream of the firefly *luciferase* gene in the pMIR-REPORT vector (Ambion, Inc.). Another vector was constructed which carried a mutation of the NF1 3′UTR in the seed region of the miR-193b binding site using the indicated primers (Table S1B). Twenty-four hours before transfection, FaDu cells were seeded in 24-well plates. Cells were then transfected with 40 nM of LNA-scrambled or LNA-193b, and 4–6 hours later, cells were co-transfected with 100 ng of pMIR-REPORT or pMIR-REPORT NF1-UTR, along with 50 ng of pRL-SV40 vector (Promega Biosciences) carrying the *Renilla luciferase* gene. At 24–72 h after transfection, luciferase activity was measured using the Dual-Glo luciferase assay system (Promega, Biosciences), with the firefly luciferase activity normalized to that of *Renilla* luciferase.

### Western Blot

FaDu cells were transfected with LNA-scrambled or LNA-193b, 72 hours post-transfection and lysed in 1 M Tris-HCl (pH 8), 5 M NaCl, and 1% NP40 plus the protease inhibitor cocktail (Roche Diagnostics). Protein concentration was assessed using the Bio-Rad Detergent-Compatible Protein Assay (Bio-Rad Laboratories). A total of 30 mg of protein was loaded onto 4–12% Tris-glycine protein gels (Invitrogen) for electrophoresis. The protein was transferred onto a PVDF (polyvinylidene fluoride) membrane using a mini Trans-Blot wet Transfer Cell (Bio-Rad). Next, membranes were blocked with Odyssey Blocking Buffer (Li-cor Biosciences). The membranes were probed with anti-NF1 rabbit polyclonal antibody (SC-67, 1/500 dilution, Santa Cruz), anti-Phospho-Erk rabbit monoclonal antibody (4370, 1/1000 dilution, Cell Signalling Technology), anti-Erk mouse monoclonal antibody (L34F12 1/1000 dilution, Cell Signalling Technology), anti-tubulin mouse monoclonal antibody (1/5000 dilution, Sigma), anti-B-actin mouse monoclonal antibody (1/5000 dilution, Sigma), all followed by IRDye(r) Fluorescent secondary antibodies (1∶20,000 dilution; Li-Cor Biosciences). Westerns blots were quantified using Odyssey Application Software 2.1 (Li-Cor Biosciences). When blotting for p-ERK at the 72-hour time point, the media was changed 24 hours after transfection with LNA-193b and LNA-scrambled since no protein otherwise would be detected.

### Immunoprecipitation

Cells were lysed in 1 M Tris-HCl (pH 8), 5 M NaCl, and 1% NP40 plus the protease inhibitor cocktail (Roche Diagnostics). Immunoprecipitations were preformed with glutathione-Sepharose 4 Fast Flow (GE Healthcare) coupled to GST-RBD (GST-fused to the Ras binding domain) [16] and then combined with lysed LNA Scramble or LNA 193b cells for 2 hours at 4°C. The mixture was then washed four times with lysis buffer containing 220 mM NaCl and eluted. Whole Cell lysates or immunoprecipitates were resolved with PVDF (polyvinylidene fluoride) membrane using a mini Trans-Blot wet Transfer Cell (Bio-Rad), as described above. Proteins were probed using anti-RAS mouse monoclonal antibody (1/1000 dilution, Santa Cruz).

### P-ERK Inhibition

FaDu cells were seeded in 6- or 96-well plates and 24 hours later, treated with 10 uM of U0126 (Cell Signalling Technology) or DMSO alone. MTS, migration and invasion assay were performed as described above.

### In situ Hybridization (ISH) of miR-193b Expression

Expression of miR-193b was evaluated in HNSCC patient samples using *in situ* hybridization on 5 um thick FFPE sections. A catalyzed signal amplification system method (GenPoint signal amplification system; DakoCytomation) was performed using 5′ biotin-labelled miR-193b miRCURY™ locked nucleic acid detection probe, or a scrambled negative control probe (50 nmol/L; Exiqon). Positive hybridization signals were visualized by adding the chromogenic substrate diaminobenzidine.

### Immunohistochemistry of P-ERK

Expression of p-ERK was evaluated on primary HNSCC sections using microwave antigen retrieval in combination with the Level-2 Ultra Streptavidin System and anti-Phospho-Erk rabbit mAB (4370, 1/600 dilution).

### Statistical Analysis

All experiments have been performed at least three independent times, and the data are presented as the mean ± SEM. The statistical significance between two treatment groups was determined using the Student’s *t* test. Analysis and graphs were completed using Graphpad Prism Software (Graphpad Software, Inc).

## Results

### miR-193b is Over Expressed in Primary HNSCC Tissues and Cell Lines

**Figure 1:**
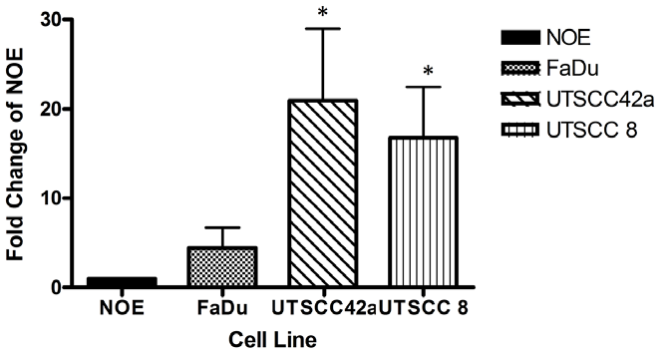
miR-193b is over expressed in HNSCC. (A) qRT-PCR analysis of miR-193b expression in FaDu, UTSCC 42a and UTSCC 8 compared with the NOE cell line. *P<0.05.

Global miRNA expression profiling of 51 primary HNSCC tissues previously conducted in our lab revealed elevated miR-193b expression in relapsed *vs*. non-relapsed patients (Fig. S1A) [10]. Similarly, over expression of miR-193b was also observed in the FaDu, UTSCC 42a and UTSCC 8 cell lines, compared to the normal oral epithelial (NOE) cell line (Fig. 1).

### *In vitro* and *in vivo* Effects of miR-193b Down Regulation

**Figure 2:**
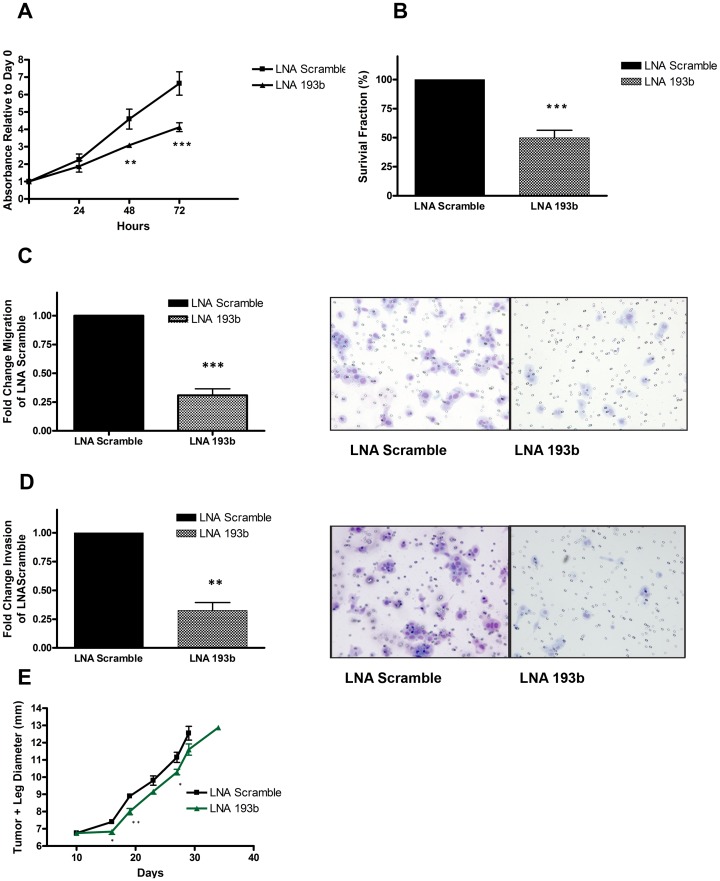
miR-193b downregulation reduced cell proliferation, migration and invasion *in vitro,* and delayed tumor formation *in vivo*. (A) FaDu cells were transfected with 40 nM of LNA-scramble or LNA-193b. Cell viability was assessed in FaDu cells by the MTS assay 24–72 hours post transfection. (B) Clonogenic survival of FaDu cells was measured 10 to 12 days after re-seeding cells treated with LNA-scramble (40 nM) or LNA-193b (40 nM) for 72 hours. (C) Representative images (right) and quantification (left) depicting the reduced ability of FaDu cells to migrate after transfection with LNA-193b (40 nM) compared to LNA-scrambled (40 nM). (D) Representative images (right) and quantification (left) depicting the reduced ability of FaDu cells to invade after transfection with LNA-193b (40 nM) compared to LNA-scrambled (40 nM). (E) FaDu cells were transfected with LNA-193b (40 nM) or LNA-scramble (40 nM) and 48 hours later implanted intramuscularly (IM) into the left gastrocemius of SCID mice. Tumor plus leg diameter was measured two to three times a week (y-axis). Data presented as mean ± SE n = 5 (LNA-scrambled), n = 6 (LNA-193b). *P<0.05, **P<0.005, ***P<0.0005, P = ns (not significant).

In order to assess the biological significance of miR-193b over expression, knockdown experiments were conducted in the HNSCC cells using a locked nucleic acid (LNA) approach. Corroboration of sustained reduction in miR-193b expression after LNA-193b was observed for up to 72 hours for all three HNSCC cell lines (Fig. S1B), with the most significant reduction observed in the FaDu cells at 72 hours. Consistent with these results, FaDu cells demonstrated a significant reduction in cell viability and colony formation (by 50%) 72 hours after transfection with LNA-193b compared to LNA-Scramble (Fig. 2A&2B); these results were replicated in the UTSCC 42 and 8 cell lines (Fig. S1C&S1D). Cell cycle analysis was employed to examine the mode of cytotoxicity inflicted by miR-193b knock-down. FaDu cells transfected with LNA-193b displayed a slight increase in the percentage of cells in sub-G1 phase with a corresponding minor reduction in the G0–G1 population (Fig. S1E). The FaDu cells were selected as the primary model for the majority of this study given their superior transfection efficiency, sustained knockdown, and consistent phenotype observed across the MTS, clonogenic and cell cycle assays.

Suppression of miR-193b also led to a significant decrease in migration (30%), and invasion (35%) of FaDu cells compared to negative control (Fig. 2C, 2D). Tumorigenicity was measured *in vivo* using SCID mice injected intra-muscularly with FaDu cells transfected with LNA-193b or LNA-scrambled. Although not significant, suppression of miR-193b delayed tumour formation compared to the negative control (Fig. 2E), which corroborated the *in vitro* miR-193b phenotype. To better understand the pathway mediating this phenotype, we sought to identify potential mRNA targets of miR-193b.

### MiR-193b is a Negative Regulator of Tumour Suppressor Genes in HNSCC

**Figure 3:**
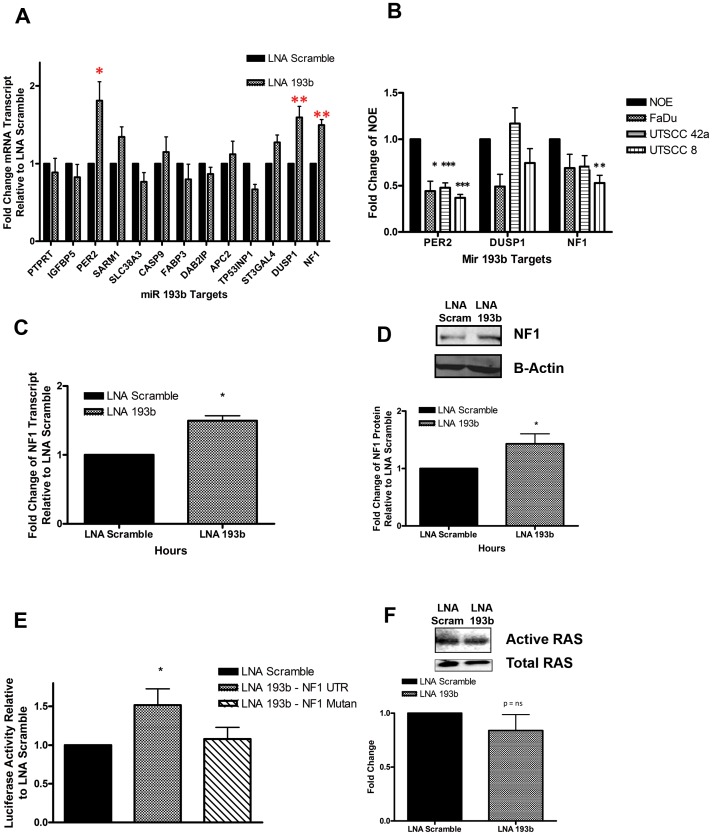
Identification of mRNA targets of miR-193b. (A) qRT-PCR of 13 predicted miR-193b targets, 72 hours post transfection with LNA-193b (40 nM), fold change relative to LNA-scramble (40 nM). (B) Basal mRNA expression of PER2, DUSP1 and NF1 was assessed using qRT-PCR in all three HNSCC cell lines relative to NOE cells. (C) NF1 transcript expression in FaDu cells was measured 72 hours post transfection with LNA-193b (40 nM) or LNA-scramble (40 nM). (D) Western blotting of NF1 in FaDu cells was determined 72 hours post transfection, images (above), quantification (below). (E) Relative luciferase activity of FaDu cells after co-transfection with pMIR-NF1 UTR (100 ng) or pMIR-NF1 Mutant (100 ng) vectors, and LNA-193b (40 nM) or LNA-scramble (40 nM), 72 hours post transfection. (F) Immunoprecipitation of GST-RBD in FaDu cells post transfection with LNA 193b (40 nM) or LNA Scramble (40 nM); images (above), quantification (below). *P<0.05, **P<0.005, ***P<0.0005, P = ns (not significant).

To identify potential targets of miR-193b, we used a tri-modality approach previously described by our lab which incorporates *in silico* prediction algorithms, experimentally- determined data, and publicly available patient data [17]; more details are provided in Fig. S2A. With this approach, 13 overlapping transcripts were identified as potential targets under of miR-193b: PRPRT, IGFBP5, PER2, SARM1, SCL38A3, CASP9, FABP3, DAB21P, APC2, TP53INP1, ST3GAL3, DUSP1 and NF1. To validate these candidate targets, mRNA transcript levels were measured by qRT-PCR at 72 hours post-miR-193b knockdown. The results demonstrated that the level of 3 candidate targets (PER2, DUSP1 and NF1) increased significantly by >1.5-fold after miR-193b knockdown, compared to the negative control (Fig. 3A). Next, the basal mRNA expression level of PER2, DUSP1 and NF1 was evaluated in the HNSCC cell lines (FaDu, UTSCC42a and UTSCC8) compared to the NOE cells, demonstrating that PER2 and NF1 were indeed significantly lower in these cell lines (<0.75 fold), thus were selected for further validation (Fig. 3B). After miR-193b knockdown, both NF1 and PER2 transcript (Fig. 3C, and Fig. S2B, S3A–S3B) and protein expression levels (Fig. 3D and Fig. S3C) increased significantly compared to the negative control. These findings indicated that both NF1 and PER2 were highly probable targets of miR-193b in HNSCC cells.

### MiR-193b Directly Targets NF1 in HNSCC Cell Lines

To establish a direct interaction between miR-193b and the 3′UTR of NF1, a luciferase assay was employed. FaDu cells co-transfected with pMIR-REPORT-NF1 and LNA-193b demonstrated a significant increase in luciferase activity (∼1.5-fold) compared to control cells (Fig. 3E). This effect was completely abrogated after mutating the NF1 3′UTR of miR-193b, thereby validating NF1 as a *bona fide* miR-193b target (Fig. 3E). Similarly, a luciferase construct was designed for PER2 but demonstrated only a weak interaction with miR-193b (Fig. S3D). Thus, NF1 was selected for further characterization based on the stronger luciferase interaction with miR-193b.

**Figure 4:**
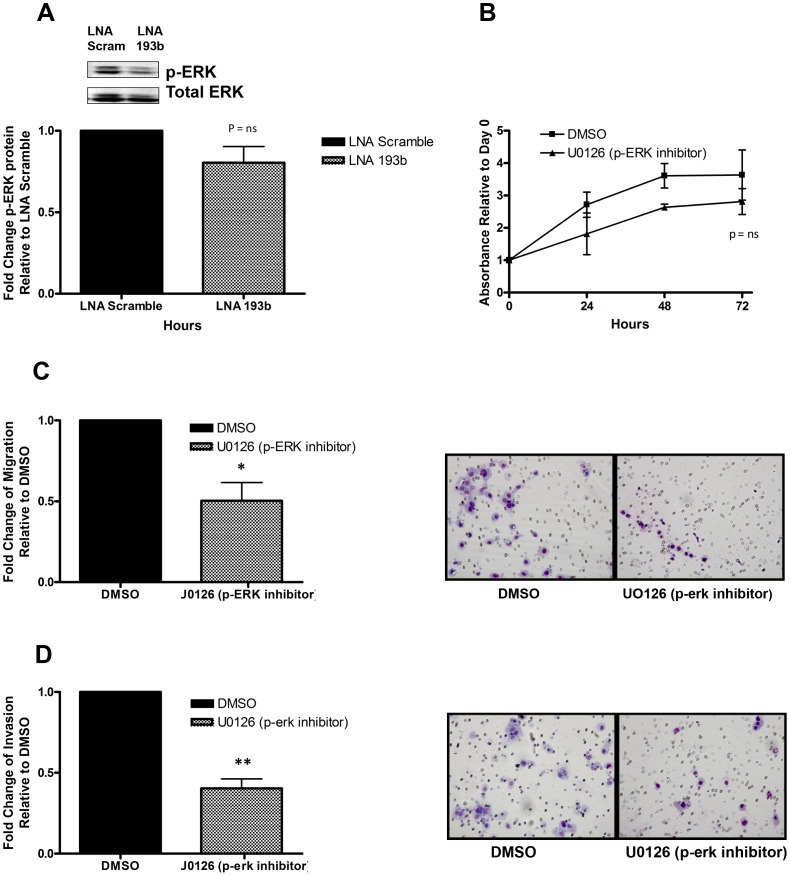
P-Erk expression contributes to cell viability, migration and invasion. Western blotting of p-ERK was measured in FaDu cells 72 hours post transfection with LNA-193b (40 nM) or LNA-scrambled (40 nM); images (above), quantification (below). (B) Cell viability of FaDu cells was assessed by the MTS assay 24, 48 and 72 hours after treatment with U0126 (10 uM) or vehicle control DMSO (10 uM). (C) Representative images (right) and quantification (left) depicting the reduced ability of FaDu cells to migrate after treatment with U0126 (10 uM) or vehicle control DMSO (10 uM). (D) Representative images (right) and quantification (left) depicting the reduced ability of FaDu cells to invade after treatment with U0126 (10 uM) or vehicle control DMSO (10 uM). *P<0.05, **P<0.005, P = ns (not significant).

NF1 is a GTPase which accelerates the conversion of active RAS-GTP into inactive RAS-GDP, thereby negatively regulating RAS signalling [18]. To determine whether the interaction between miR-193b and NF1 affected RAS signalling, we performed an immunoprecipitate using GST-RBD, to assess changes in active RAS. Knockdown of miR-193b in FaDu cells led to a modest decrease in active RAS compared to the negative control (Fig. 3F). To examine if the interaction between miR-193b and NF1 propagated down the RAS signalling pathway, we evaluated p-ERK expression, a kinase located downstream of RAS. Knockdown of miR-193b in FaDu cells indeed led to a decrease in p-ERK expression compared to the negative control (Fig. 4A). Given the involvement of p-ERK in promoting cell proliferation and migration, we treated FaDu cell with a MEK/ERK kinase 1/2 pharmacologic inhibitor (U1026) which blocks p-ERK activity [19]. FaDu cells treated with U0126 demonstrated a reduction in cell viability compared to the negative control (DMSO) (Fig. 4B). Furthermore, FaDu cells treated with U0126 illustrated a significant reduction in migration (50%) and invasion (45%) compared to cells treated with DMSO (Figs. 4C&D). These results recapitulated the phenotype observed after miR-193b knockdown, suggesting that this pathway might indeed be mediating the effects of the miR-193b∼NF1 axis.

### MiR-193b Over Expression is Associated with Poor Survival and Increased p-ERK Expression in Primary HNSCC

**Figure 5:**
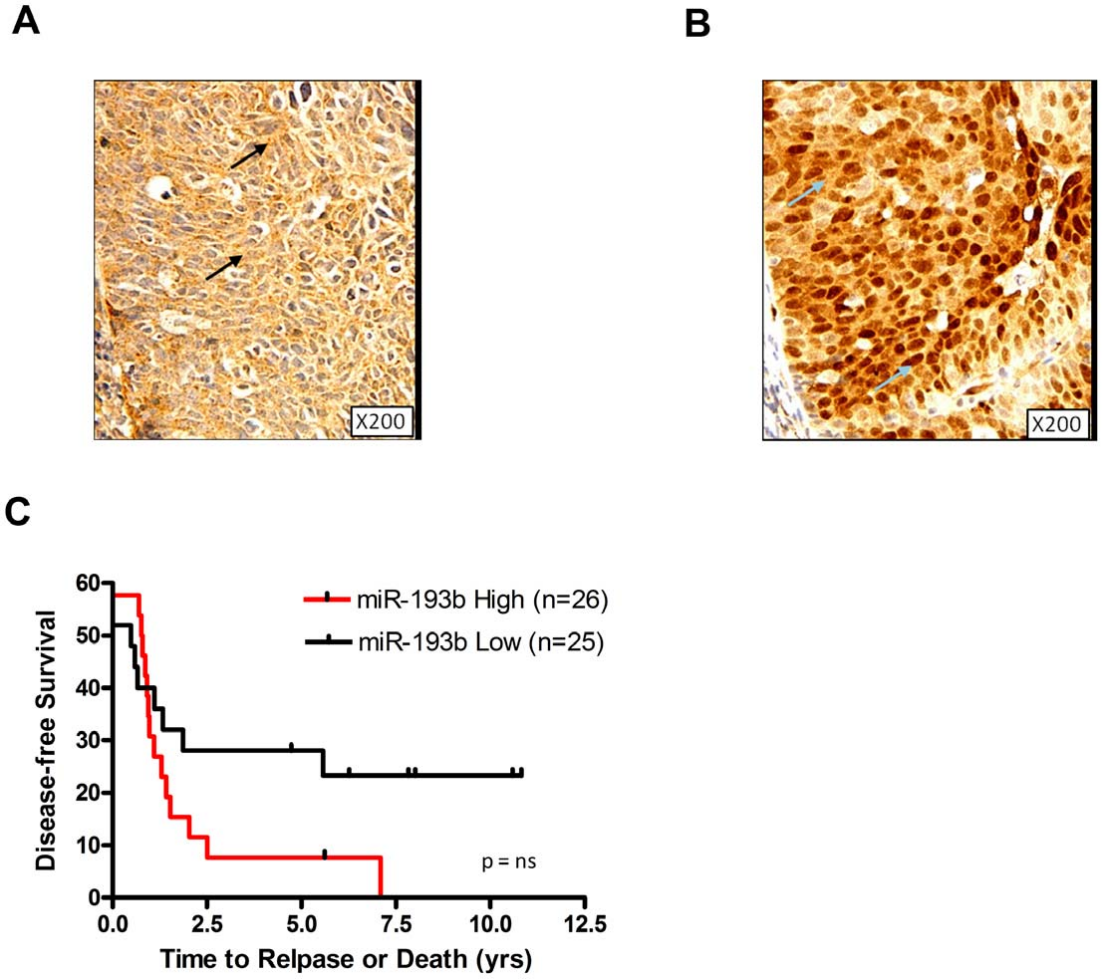
MiR-193b and p-ERK expression in a primary HNSCC patient sample. (A) A representative image of miR-193b *in situ* hybridization of a primary HNSCC biopsy, arrows indicating tumor cells exhibiting cytoplasmic signal. (B) A representative image of immunohistochemical analysis of p-ERK expression in the same patient’s HNSCC biopsy sample; arrows indicating expression in both the tumor nucleus and the cytoplasm. (C) Kaplan-Meier plots of disease free survival for HNSCC patients dichotomized based on high (>median) *vs*. low (≤ median) miR-193b expression. P = ns (not significant).

*In situ* hybridization was utilized to visually confirm miR-193b expression in primary HNSCC tissues. A miR-193b signal was observed in the cytoplasm of the tumour cells (Fig. 5A), but not in the adjacent infiltrating lymphocytes, or when using the scrambled control probe (Figs. S4A–B). Another negative control was provided by a primary human breast cancer tissue wherein no miR-193b signal was observed (Fig. S4C). In the same HNSCC biopsy tissue, intense immuno-expression of p-ERK was also observed in the nucleus and cytoplasm of the tumour cells, but not in the adjacent stroma or infiltrating lymphocytes (Fig. 5B, and Fig. S4D). These findings support an association between miR-193b with p-ERK co-expression. Finally, when the expression of miR-193b from the 51 HNSCC patients previous profiled by our lab [10] was dichotomized between high miR-193b (> median) *vs*. low (≤ median) expression, the former group experienced a worse disease-free survival compared to the latter (HR = 1.41; p = 0.18); although statistical significance was not attained due to the small cohort size (Fig. 5C).

## Discussion

**Figure 6:**
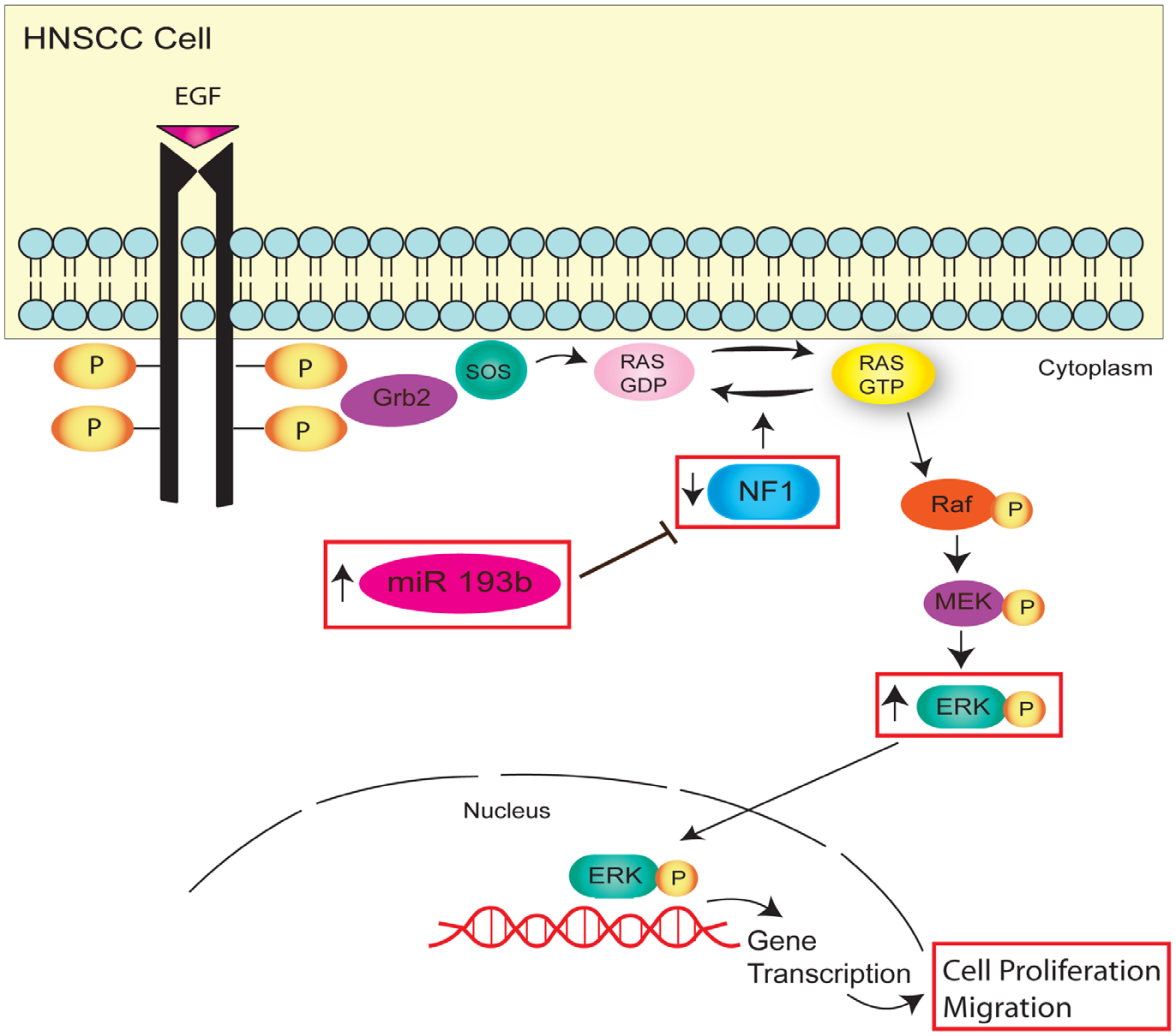
Proposed model for the role of miR-193b in regulating HNSCC cell proliferation and migration. Schema showing over expression of miR-193b leads to the down regulation of NF1, which prevents RAS-GTP from hydrolyzing into inactive RAS-GDP. This in turn triggers RAS signalling which leads to activation of a cascade of mitogen activated protein kinases (MAPKs), including p-ERK which can then translocate into the nucleus and promotes the expression of genes involved in cell proliferation and migration, driving HNSCC progression. Boxes indicate data demonstrated in this study.

A global miRNA profiling study previously conducted in our lab [10] identified the over expression of miR-193b as a dysregulated miRNA in HNSCC patients. Herein, we report for the first time that miR-193b can target NF1, which in turn leads to ERK activation, as one pathway to promote HNSCC progression (Fig. 6). Our study demonstrated that the down regulation of miR-193b decreased cell viability, clonogencity, migration, invasion, and tumour formation, which in turn, was associated with increased NF1 and a decrease in ERK phosphorylation. Furthermore, HNSCCs with higher miR-193b expression levels fared worse than lower miR-193b tumours, which all collectively demonstrate that dysregulation of the miR-193b∼NF1∼ERK axis is yet another mechanism which can drive HNSCC progression.

Over expression of miR-193b has been described previously in HNSCC [11], uveal melanomas [20] and multiple myeloma [21]. The precise mechanism for miR-193b over-expression remains unclear; it is located on chromosome 16p11.13, a region not previously described to be amplified in HNSCC. Alterations in key players of the miRNA processing machinery, such as Dicer, Drosha, DGCR8, AGO2 or XPO5, have been reported to contribute to aberrant miRNA expression [22]. For instance, elevated global miRNA expression in salivary gland pleomorphic adenomas have been associated with elevated expression of Dicer, Drosha or DGCR8 expression [23]. In addition, oncogenic proteins such as MYC have been described to bind to the locus of miRNAs such as miR-9, activating their transcription [24]. Hence, it is certainly possible that several different mechanisms can account for elevated miR-193b expression in HNSCC, although the precise process remains to be elucidated.

The role of miR-193b in human malignancies appears to be controversial. In our study, miR-193b appears to be mediating oncogenic signals; other reports in breast, prostate and leukemia however, have identified miR-193b to be a tumour suppressor, wherein low miR-193b expression promoted tumour progression [25], [26], [27]. The opposing effects of the same miRNA in different malignancies have been previously reported. As an example, miR-205, which was observed to be over expressed in HNSCC [10], [28], [29], endometrial [30] and lung [31] cancers, was in fact under-expressed in breast cancers [32]. Similarly, global miRNA profilings of six human solid malignancies demonstrated tissue specific expression of miRNAs [33], further corroborating the complex expression patterns and biology of miRNAs.

In this current study, we identified NF1 as one target for miR-193b. NF1 is a RAS-GTPase (see Fig. 6), which accelerates the conversion of active RAS-GTP into inactive RAS-GDP, thereby negatively regulating RAS signalling [34]. RAS bound to GTP activates Raf which triggers a cascade of mitogen activated protein kinases (MAPKs). Signals emanating from this cascade can regulate genes involved in cell growth, death, differentiation and migration [18], demonstrating the central role for this pathway in promoting tumour progression. In glioblastoma, NF1 inactivating mutations were identified from a large panel of sequenced tumors, suggesting NF1’s relevance to the development of sporadic glioblastoma [35], [36]. Furthermore, mutations and genomic alterations of NF1 have been reported in a number of other cancer cells and tumor tissues [37], [38], [39]. *In vitro*, the importance of NF1 was demonstrated in NIH 3T3 cells, which when over expressed, displayed a reduction in cell growth compared to untreated cells [40], further supporting a tumor suppressor role for NF1 *via* RAS inhibition.

ERK is a MAPK kinase located downstream of Raf and MEK in the RAS signalling pathway. We observed an inverse relationship between NF1 and ERK activation, whereby suppression of miR-193b led to an increase in NF1 and decreased p-ERK. These findings illustrate the ability of the miR-193b∼NF1 interaction to propagate downstream of RAS signalling. Since activated p-ERK can translocate into the nucleus and bind to the promoter of genes which promote cell growth, death and migration [18], [41], it follows that the observed miR-193b phenotype might be attributable to ERK activation. This was corroborated by pharmacologic inhibition of p-ERK (with U0126), which recapitulated the same phenotype as observed with miR-193b knockdown. Moreover, HNSCC patients with high miR-193b expression also expressed high levels of p-ERK, providing a translational corroboration between miR-193b and p-ERK. The relevance of the ERK pathway in HNSCC has also been reported by others, wherein treatment of HNSCC cells (UM-SCC-9 and UM-SCC-11B) with U0126 decreased cell viability [42], confirming the observations made in this current study.

Given the negative regulatory role of NF1 on RAS activation, it is conceivable that tumours which over-express miR-193b, thereby down regulating NF1, could constitutively activate the RAS signalling pathway. Colorectal cancer patients with activating RAS mutations are resistant to EGFR inhibitors such as Cetuximab [43], [44]. The landmark trial of Cetuximab combined with radiation therapy [45] has transformed clinical management of HNSCC; however, not every patient responds to this regimen, suggesting alternate pathways by which HNSCCs can escape EGFR inhibition [46]. One speculation that could arise from our data might be the possibility that the miR-193b∼NF1 axis might account for Cetuximab resistance. Thus, it would be of interest to assay miR-193b expression in relation to Cetuximab sensitivity in HNSCC patients.

PER2 was the second potential miR-193b target identified in this study; PER2 is a tumour suppressor involved in regulation of the circadian rhythm, which in turn controls cellular processes such as proliferation, apoptosis, metabolism and DNA repair [47]. In this current study, we established an interaction between miR-193b and PER2, whereby miR-193b knockdown increased PER2 transcript and protein expression (Fig. S3). PER2 under-expression was recently reported for 40 HNSCC primary patient tumour samples [48]; furthermore, leukemic cells over expressing PER2 also led to a reduction in proliferation and clonogenicity [49], which corroborates our miR-193b phenotype (Fig. 2). Finally, PER2 has also been described to be a negative regulator of c-myc [50], a potent oncogene involved in proliferation, differentiation, migration, and invasion [51]. Hence, in this current report, we propose a miRNA-mediated mechanism for PER2 under-expression, which in turn, promotes HNSCC progression.

The translational impact of this study have been demonstrated by the potential prognostic value of miR-193b over expression in its association with worse outcome (Fig. 5C), in turn associated with P-ERK expression (Fig. 5B). Micro-RNAs themselves cannot yet readily serve as therapeutic targets; however, it is conceivable that the ERK pathway could be potentially inhibited. Pre-clinical studies have certainly demonstrated the growth inhibitory effects of p-ERK inactivation in HNSCC cells [42], along with reduced migration and invasion. There are medicinal chemistry approaches currently being explored for more specific ERK inhibitors [52], [53], [54]; our present study would certainly suggest this could indeed be a fruitful pursuit in an effort to further improve outcome for HNSCC patients, particularly those who might be resistant to Cetuximab combined with RT.

In summary, we have identified a novel oncogenic role for miR-193b, whereby high miR-193b suppresses NF1, in turn activating p-ERK, leading to increased HNSCC cell proliferation, invasion, migration, and tumour formation (Fig. 6). These findings suggest yet another mechanism which could account for the aggressive behaviour of this disease. The translational impact of this study lie in the potential prognostic value of miR-193b, along with P-ERK serving as a possible therapeutic target by which outcome for HNSCC patients could be further improved.

## Supporting Information

Figure S1 **miR-193b over expression in HNSCC induced cell proliferation in UTSCC 42a and UTSCC 8 cells.** (A) qRT-PCR analysis for miR-193b was conducted on 51 HNSCC primary tissue samples showing high expression in 43 relapsed vs. low expression for 8 non- relapsed tumors, relative to the expression of four normal larynx tissues. (B) qRT-PCR of miR-193b expression in HNSCC cell lines 24–72 hours after transfection with LNA-193b (40 nM) or LNA-scramble (40 nM). (C) Cell viability was assessed in UTSCC 42a and 8 cells by the MTS assay 24, 48 and 72 hours post transfection with LNA-193b (40 nM) or LNA-scramble (40 nM). (D) Clonogenic survival of UTSCC 42a and 8 cells was measured 10 to 12 days after transfection with LNA-193b (40 nM) or LNA-scramble (40 nM). (E) Cell cycle analysis was performed on FaDu cells using flow cytometry 72 hours post transfection with LNA-193b (40 nM) or LNA-scramble (40 nM). **P<0.005, ***P<0.0005, P = ns (not significant).
(TIF)

Figure S2 **Identification of mRNA targets of miR-193b.** (A) Venn diagram showing the tri-modality approach used to identify miR-193b targets. (B) NF1 transcript expression of UTSCC 42a and 8 cells was measured 72 hours post transfection with LNA-193b (40 nM) or LNA-scramble (40 nM). P = ns (not significant).
(TIF)

Figure S3 **Identification of PER2 as a target of miR-193b.** (A) PER2 transcript expression in FaDu cells was measured 72 hours post transfection with LNA-193b (40 nM) or LNA-scramble (40 nM). (B) PER2 transcript expression in UTSCC 42a was measured 72 hours post transfection with LNA-193b (40 nM) or LNA-scramble (40 nM). (C) Western blotting of PER2 in FaDu cells lines was determined 72 hours post transfection, images (above), quantification (below). (D) Relative luciferase activity of FaDu cells after co-transfection with pMIR-PER2 UTR (100 ng) or pMIR-PER2 Mutant (100 ng) vectors with LNA-193b (40 nM) or LNA-scramble (40 nM). *P<0.05, **P<0.005, P = ns (not significant).
(TIF)

Figure S4 **MiR-193b targets the RAS signalling pathway** ***in vitro*** **and across HNSCC patient samples.** (A) A representative image of miR-193b *in situ* hybridization of primary HNSCC biopsy samples, arrows indicate tumor cells exhibiting cytoplasmic staining. (B) Representative image of control *in situ* hybridization of primary HNSCC biopsy samples using a scramble probe. (C) Representative image of miR-193b *in situ* hybridization of primary breast cancer sample. (D) Representative image of immunohistochemical analysis of p-ERK expression in primary HNSCC biopsy samples (same patient as A), arrows indicate tumors exhibiting nuclear and cytoplasmic staining.
(TIF)

Table S1 (A) qRT-PCR primer design sequences (B) Cloning primer design sequences.
(TIF)
