# Supplementary figures and images for: MicroRNA-193b Enhances Tumor Progression via Down Regulation of Neurofibromin 1
Source: PLoS One. 2013 Jan 15;8(1):e53765. doi: 10.1371/journal.pone.0053765 (PMC3546079; doi:10.1371/journal.pone.0053765)

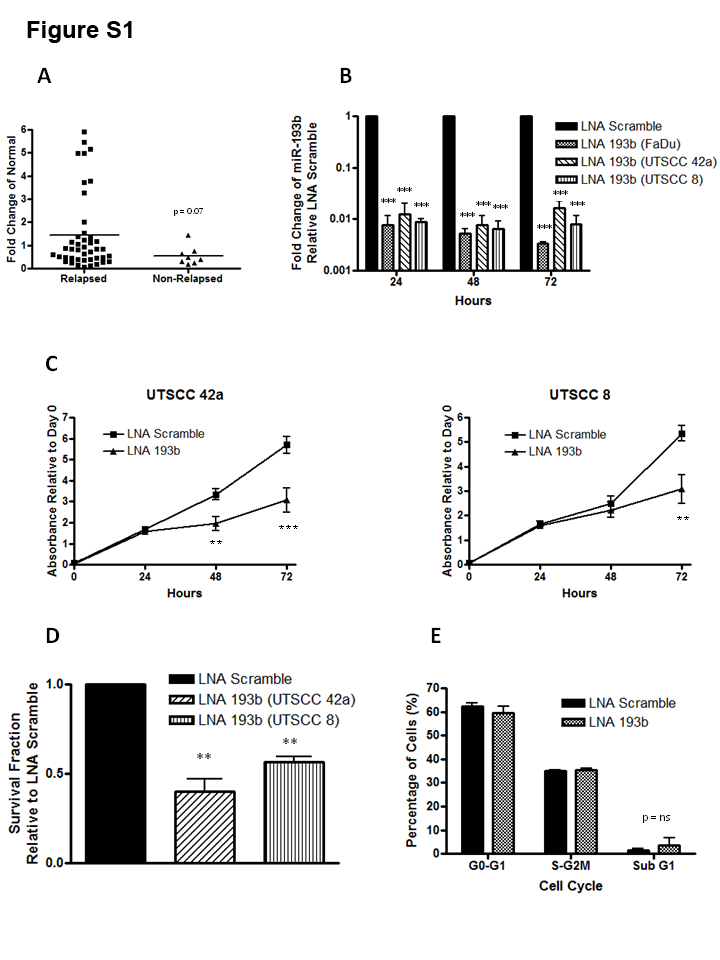

Supplement: Figure S1 — miR-193b over expression in HNSCC induced cell proliferation in UTSCC 42a and UTSCC 8 cells. (A) qRT-PCR analysis for miR-193b was conducted on 51 HNSCC primary tissue samples showing high expression in 43 relapsed vs. low expression for 8 non- relapsed tumors, relative to the expression of four normal larynx tissues. (B) qRT-PCR of miR-193b expression in HNSCC cell lines 24–72 hours after transfection with LNA-193b (40 nM) or LNA-scramble (40 nM). (C) Cell viability was assessed in UTSCC 42a and 8 cells by the MTS assay 24, 48 and 72 hours post transfection with LNA-193b (40 nM) or LNA-scramble (40 nM). (D) Clonogenic survival of UTSCC 42a and 8 cells was measured 10 to 12 days after transfection with LNA-193b (40 nM) or LNA-scramble (40 nM). (E) Cell cycle analysis was performed on FaDu cells using flow cytometry 72 hours post transfection with LNA-193b (40 nM) or LNA-scramble (40 nM). **P<0.005, ***P<0.0005, P = ns (not significant). (TIF) [file pone.0053765.s001.tif]

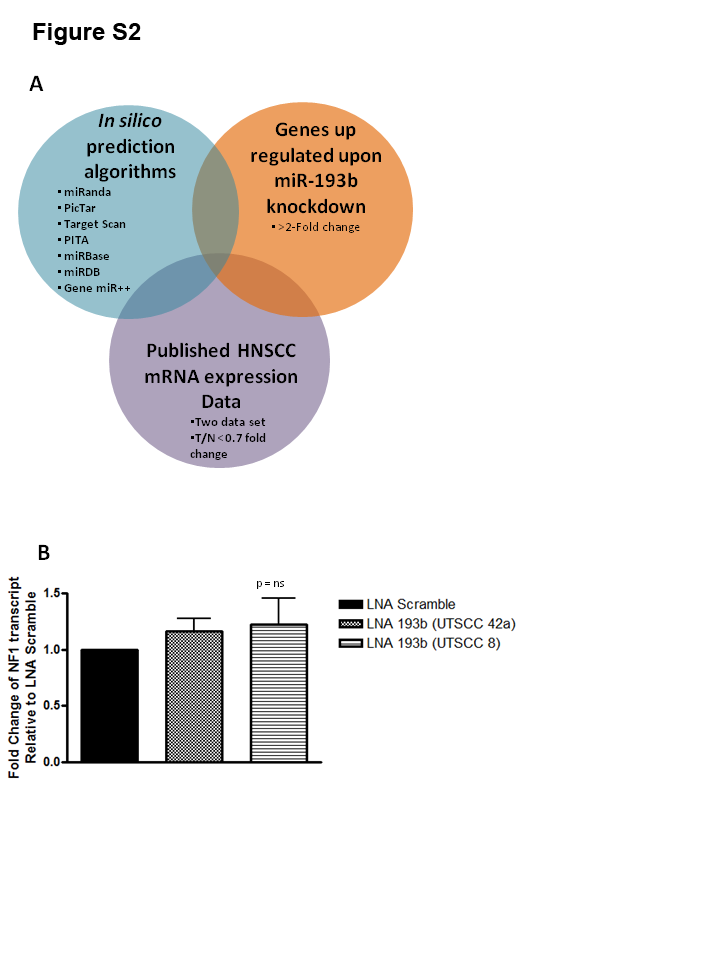

Supplement: Figure S2 — Identification of mRNA targets of miR-193b. (A) Venn diagram showing the tri-modality approach used to identify miR-193b targets. (B) NF1 transcript expression of UTSCC 42a and 8 cells was measured 72 hours post transfection with LNA-193b (40 nM) or LNA-scramble (40 nM). P = ns (not significant). (TIF) [file pone.0053765.s002.tif]

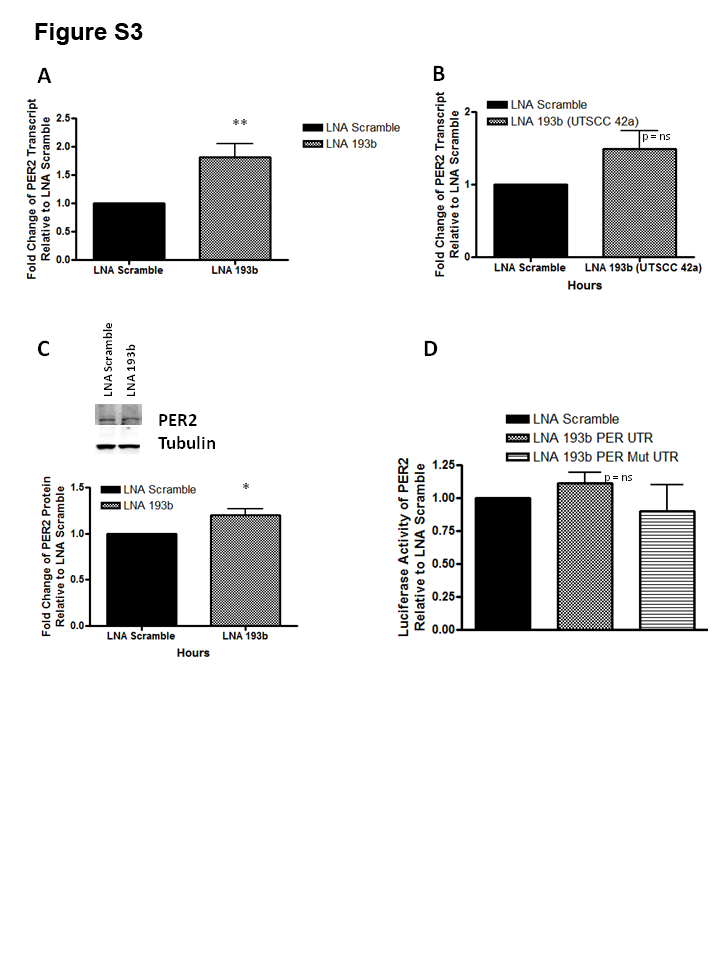

Supplement: Figure S3 — Identification of PER2 as a target of miR-193b. (A) PER2 transcript expression in FaDu cells was measured 72 hours post transfection with LNA-193b (40 nM) or LNA-scramble (40 nM). (B) PER2 transcript expression in UTSCC 42a was measured 72 hours post transfection with LNA-193b (40 nM) or LNA-scramble (40 nM). (C) Western blotting of PER2 in FaDu cells lines was determined 72 hours post transfection, images (above), quantification (below). (D) Relative luciferase activity of FaDu cells after co-transfection with pMIR-PER2 UTR (100 ng) or pMIR-PER2 Mutant (100 ng) vectors with LNA-193b (40 nM) or LNA-scramble (40 nM). *P<0.05, **P<0.005, P = ns (not significant). (TIF) [file pone.0053765.s003.tif]

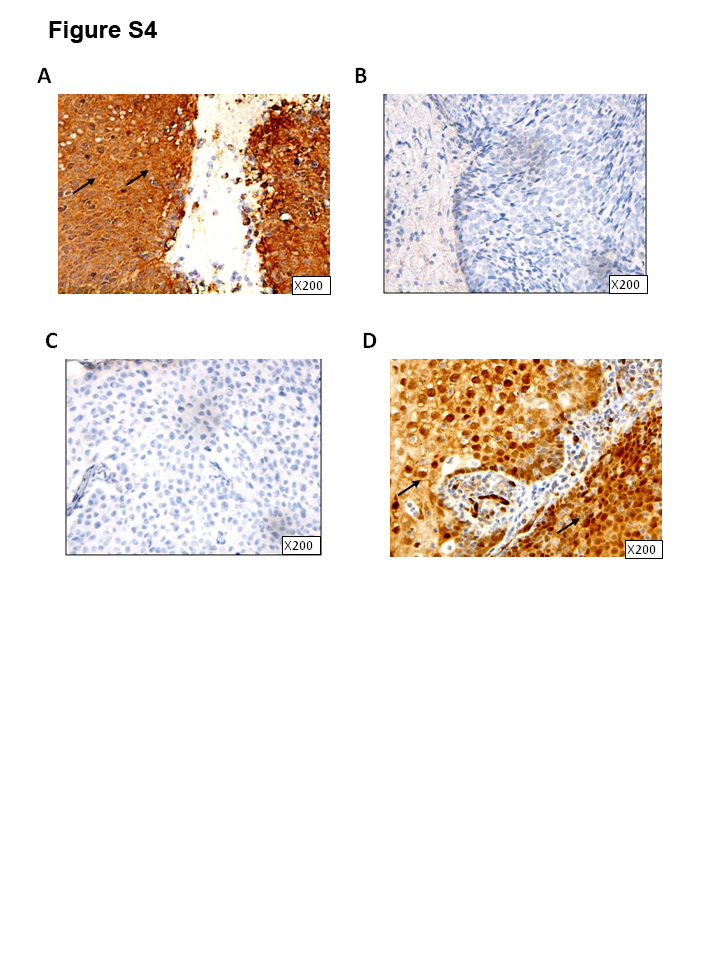

Supplement: Figure S4 — MiR-193b targets the RAS signalling pathway in vitro and across HNSCC patient samples. (A) A representative image of miR-193b in situ hybridization of primary HNSCC biopsy samples, arrows indicate tumor cells exhibiting cytoplasmic staining. (B) Representative image of control in situ hybridization of primary HNSCC biopsy samples using a scramble probe. (C) Representative image of miR-193b in situ hybridization of primary breast cancer sample. (D) Representative image of immunohistochemical analysis of p-ERK expression in primary HNSCC biopsy samples (same patient as A), arrows indicate tumors exhibiting nuclear and cytoplasmic staining. (TIF) [file pone.0053765.s004.tif]

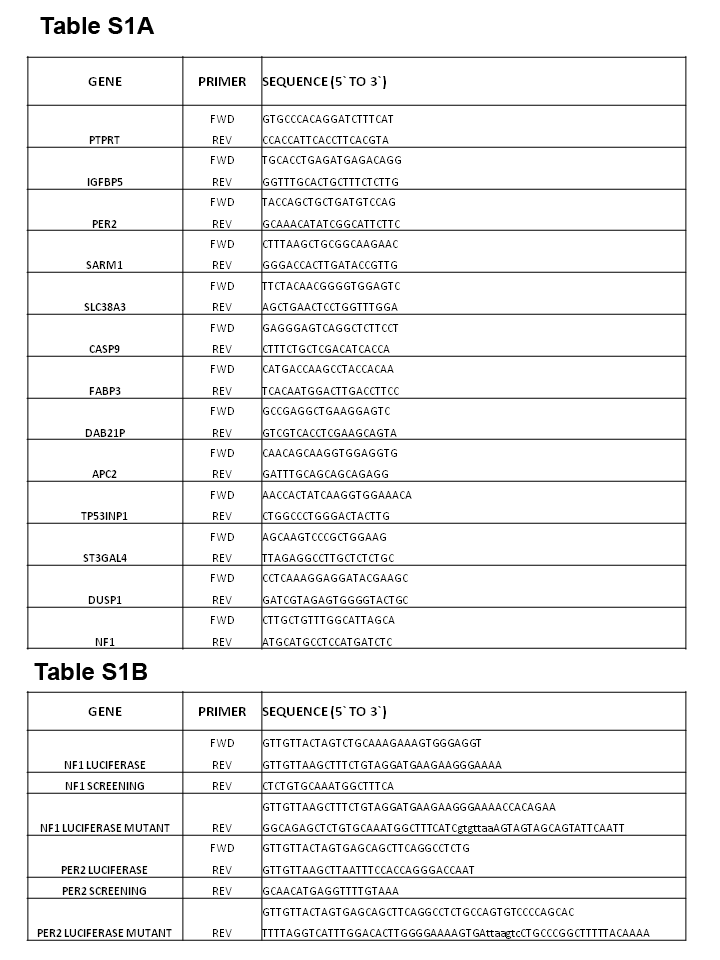

Supplement: Table S1 — (A) qRT-PCR primer design sequences (B) Cloning primer design sequences. (TIF) [file pone.0053765.s005.tif]
